# Exploration of Redox-Related Molecular Patterns and the Redox Score for Prostate Cancer

**DOI:** 10.1155/2021/4548594

**Published:** 2021-11-11

**Authors:** Yue Wu, Xi Zhang, Huan Feng, Bintao Hu, Zhiyao Deng, Chengwei Wang, Bo Liu, Yang Luan, Yajun Ruan, Xiaming Liu, Zhuo Liu, Jihong Liu, Tao Wang

**Affiliations:** ^1^Department of Urology, Tongji Hospital, Tongji Medical College, Huazhong University of Science and Technology, Wuhan, 430030 Hubei, China; ^2^Institute of Urology, Tongji Hospital, Tongji Medical College, Huazhong University of Science and Technology, Wuhan, 430030 Hubei, China; ^3^The First Clinical Medical College of Anhui Medical University, Hefei, 230001 Anhui, China; ^4^Department of Oncology, Tongji Hospital, Tongji Medical College, Huazhong University of Science and Technology, Wuhan, 430030 Hubei, China

## Abstract

Redox homeostasis is the key to cell survival, and its imbalance can promote the occurrence and progression of tumors. However, it remains unclear whether these redox-related genes (RRGs) have potential roles in the tumor microenvironment, immunotherapy, and drug sensitivity. Here, we performed a systematic and comprehensive analysis of 489 prostate cancer (PC) samples from The Cancer Genome Atlas database and 214 PC samples from 8 datasets in the Gene Expression Omnibus database to determine redox modification patterns and the redox scoring system for PC. We identified two modification patterns (Redox_A and Redox_B) in PC using unsupervised consensus clustering based on 1410 differential expression RRGs. We then compared the prognostic value, tumor microenvironment characteristics, immune cell infiltration, and molecular characteristics of the two patterns. The Redox_A pattern was significantly enriched in the carcinogenic activation signaling pathways and had a poor prognosis, while the Redox_B pattern was mainly enriched in a variety of metabolic and redox pathways and had a good prognosis. Next, redox-related characteristic genes were extracted from these two patterns, and a scoring system (Redox_score) was constructed to evaluate PC patients. Further analysis indicated that lower Redox_score patients had a better prognosis, while higher Redox_score patients had a higher tumor mutation burden, driver gene mutation rate, and immune checkpoint inhibitor gene expression. We also found that higher Redox_score patients were more responsive to anti-PD-1 immunotherapy. Moreover, Redox_score was determined to be significantly correlated with anticancer drug sensitivity and resistance. Our study provides a comprehensive analysis of redox modifications in PC and reveals new patterns of PC based on RRGs, which will provide insights into the complex mechanisms of PC and develop more effective individualized therapeutic strategies.

## 1. Introduction

Prostate cancer (PC) is the most common noncutaneous malignancy in men worldwide and the second leading cause of tumor-related death in men [1]. It was estimated that there were 191,930 new diagnoses and 33,330 deaths from PC in the United States in 2020 [1]. Distant metastasis occurs in approximately 20% of all PC patients and is the leading cause of PC-related death [2]. And the 5-year survival rate for these patients dropped significantly, to nearly 30% [3, 4]. The most common site of distant metastasis from PC is the bone, followed by the lung, lymph nodes, and liver, which are the most deadly sites of metastasis [5]. Thus, a better understanding of the occurrence and progression of PC may contribute to effective early diagnosis and targeted therapy.

Redox homeostasis is the balance of the equivalent of reduction and oxidation and has a great importance in many physiological and pathological processes. The imbalance of redox homeostasis is mainly caused by changes in reactive oxygen species (ROS)/reactive nitrogen (RNS) or antioxidant protein levels [6]. ROS are present in the cell as free radicals (OH^−^), neutral molecules (H_2_O_2_), or ions (O_2_^−^), while cellular RNS are present in the form of peroxynitrite (ONOO^−^), nitric oxide (NO), and nitrogen dioxide (NO_2_) [7, 8]. Studies have shown that RNS and ROS are crosstalk and have obvious correlation [9]. Under physiological conditions, various biological processes such as cell proliferation, cell differentiation, and adaptive immunity, as well as a variety of proteins including kinases, receptors, transcription factors, and ion channels, are dependent on ROS regulation and modification [10, 11]. However, sustained increases in intracellular ROS levels can cause a variety of pathological processes, such as cardiovascular disease, neurodegeneration, immune system dysfunction, and cancer [12]. Therefore, it is necessary to study the redox state in tumor cells.

Currently, there are not enough indicators to diagnose PC at an early stage and to distinguish between those who need a prostatectomy and those who need tumor treatment. Even prostate-specific antigen (PSA) levels, the most common marker for PC development and progression, can remain within normal ranges or have false negative results [13]. To this end, several researchers have sought to identify specific biomarkers in the redox system to determine the severity of prostate cancer. For example, the Süle et al. [13] study found that patients with early stage PC had significantly lower levels of cytokines and growth factors than controls. Blázovics et al. [14] found that the results of binding formaldehyde, Zn-protoporphyrin, and free protoporphyrin in erythrocytes were significantly different in patients with metastatic, histologically negative, and histologically positive PC treated with taxane compared with healthy controls. These findings are closely related to the redox state [15]. However, the large number of redox regulatory factors makes it difficult for traditional research methods to reflect the macrolandscape of the redox state of individual tumors. Moreover, the occurrence and progression of tumors are highly coordinated interactions of multiple regulatory factors, so a more comprehensive and effective analysis of the characteristics of redox reactions in PC is required. Herein, we comprehensively evaluated genomic changes and redox patterns by integrating transcriptome data of 489 PC samples from The Cancer Genome Atlas (TCGA) database and 214 PC samples from 8 datasets in the Gene Expression Omnibus (GEO) database. Two PC subclasses, Redox_A and Redox_B, were identified based on redox-related genes (RRGs) and by the unsupervised clustering method. Further analysis showed that the two patterns were enriched in different biological pathways and showed different characteristics of the immune microenvironment. Next, we extracted the redox-associated characteristic genes from these two modification patterns and constructed a scoring scheme (Redox_score) to quantify the redox patterns of individual tumors and evaluate its prognostic value, clinical characteristics, drug sensitivity, and immunotherapy.

## 2. Materials and Methods

### 2.1. Collection and Pretreatment of PC Datasets

Transcriptome data and corresponding clinical characteristics of PC patients were collected and collated from TCGA and GEO databases, respectively. Specifically, in the TCGA database, we mainly collected RNA sequencing data (FPKM format and read counts), somatic mutation data (MAF format), miRNA sequencing data, and clinical prognosis information of PC patients (https://portal.gdc.cancer.gov/). The FPKM format was then converted into transcripts per kilobase million (TPM) format for subsequent analysis. Next, we obtained 8 datasets from the GEO database (https://www.ncbi.nlm.nih.gov/geo/) that shared the same microarray sequencing platform (Affymetrix GPL570-HG-U133 plus 2.0), including GSE69223 (*n* = 30), GSE55945 (*n* = 19), GSE46602 (*n* = 50), GSE45016 (*n* = 11), GSE26910 (*n* = 12), GSE17951 (*n* = 154), GSE17906 (*n* = 25), and GSE3325 (*n* = 19). Subsequently, background adjustment and quantile normalization were performed on the original “CEL” files of the above 8 datasets through the “RMA” algorithm of the “affy” R package. The batch effects of merging 8 datasets were then removed by using the “ComBat” approach of the “SVA” R package. Moreover, we directly downloaded the standardized sequencing data and corresponding clinical prognosis information of the GSE70769 dataset from the GEO database and were used for subsequent score validation.

### 2.2. Differential Expression of RRGs and Identification of PC Subclasses

In order to obtain all RRGs, we used “redox” as the key word to screen human genes related to redox from the OMIM database (https://www.oncomine.org/resource/), gene function module of the NCBI database (https://www.ncbi.nlm.nih.gov/gene/), GeneCards database (https://www.genecards.org/), and GSEA-MSigDB (https://www.gsea-msigdb.org/gsea/msigdb), and finally, we got a total of 4087 RRGs. Subsequently, the differential expression RRGs were obtained through the “DESeq2” R package in TCGA-PRAD and GEO-PRAD cohorts based on the standard *P* < 0.05. 2616 differentially expressed RRGs were obtained from TCGA-PRAD and 1850 differentially expressed RRGs were obtained from GEO-PRAD. Finally, the same differentially expressed RRGs (*n* = 1410) in TCGA-PRAD and GEO-PRAD cohorts were selected for subsequent analysis. Next, we used the *k*-means algorithm in the “Consensus-Clusterplus” R package to perform unsupervised consensus clustering for these RRGs and repeated it for 1000 times to ensure classification stability [16].

### 2.3. Gene Set Variation Analysis (GSVA)

We first downloaded the “c2.cp.kegg.v7.2.symbols” gene set and “h.all.v7.4.symbols” gene set from MSigDB database. We performed GSVA analysis and differential analysis using the “GSVA” and “Limma” R packages, respectively, to explore the biological processes that significantly differed between redox patterns. In addition, we also utilized the “clusterProfiler” R package for functional annotation of these RRGs.

### 2.4. Evaluation of Immune Cell Infiltration between PC Patterns

The cell-type identification by estimating relative subsets of RNA transcripts (CIBERSORT) algorithm developed by Newman et al. [17] and its attached LM22 gene set were used to assess differences in immune cell infiltration between different PC patterns. Here, in order to make the results more reliable, 1000 permutation tests were performed, and the results were screened according to the *P* < 0.05 criterion.

### 2.5. Construction of the Redox_Score to Evaluate Individual PC

We developed a redox scoring scheme based on the genes most associated with prognosis to assess individual PC patients. Specifically, we performed differential expression analysis for PC patterns and screened the results based on ∣log_2_ fold change (FC) | >1 and adjusted *P* < 0.05. Next, univariate Cox regression analysis, least absolute shrinkage and selection operator (LASSO) regression analysis, and multivariate Cox regression analysis were performed to identify RRGs most associated with prognosis. Then, we used the regression coefficients obtained from the multivariate Cox analysis and calculated the redox score based on the following formula: Redox_score = ∑_*i*=1_^*n*^Exp*iβi*, where Exp represents the expression value of the gene and *β* represents the regression coefficient of the corresponding gene.

### 2.6. miRNA-RRG Regulatory Network and PC Mutation Analysis

miRNA expression data in PC were obtained from the TCGA database, and the differential expression miRNAs were identified between the normal group and the tumor group. We then conducted the coexpression analysis of these miRNAs and prognostic-related RRGs. miRNAs with ∣Cor | >0.3 and *P* < 0.001 were considered to be correlated. Next, the “maftools” R package was used to analyze the somatic mutation data and calculate the tumor mutation burden (TMB). We further analyzed the difference of TMB expression in different risk groups and its correlation with Redox_score. We also delineate the landscape of driving gene mutations between low- and high-risk groups.

### 2.7. Benefit of Redox_Score in Predicting Immunotherapy Reactivity

We first compared the expression differences of different immune checkpoint inhibitor (ICI) genes between low- and high-risk groups and further investigated whether the Redox_score still had an impact on the prognosis of patients when the expression of the ICI gene was considered. Then, based on available data for melanoma patients receiving immunotherapy, we analyzed the similarity of gene expression profiles between patients in different risk groups and melanoma patient groups by SubMap to indirectly predict the response of Redox_score-based PC patients to immunotherapy [18]. Moreover, we obtained an open access immunotherapy cohort of patients with metastatic melanoma undergoing anti-PD-1 therapy and performed a Kaplan–Meier analysis of pretreatment patients based on the Redox_score.

### 2.8. Correlation Analysis between Redox_Score and Drug Sensitivity

We downloaded transcriptional data of tumor cell lines, IC50 values of antitumor drugs, and drug targets/pathways from the Genomics of Drug Sensitivity in Cancer (GDSC, https://www.cancerrxgene.org/) database. Then, we did the Pearson correlation analysis between the Redox_score and drug sensitivity and according to the *P* < 0.05 and ∣Rs | >0.15 filtering results.

### 2.9. Real-Time Quantitative Polymerase Chain Reaction (RT-QPCR) Verification

We first used a TRIzol reagent (Beyotime, Jiangsu, China) to extract total RNA from various prostate cancer cells. These total RNA were then reversely transcribed into cDNA using the Hifair®III 1st Strand cDNA Synthesis SuperMix for qPCR (gDNA digester plus) (YEASEN, Shanghai, China), which was subsequently detected by qPCR using the Hieff® qPCR SYBR® Green Master Mix (Low Rox) (YEASEN, Shanghai, China) an ABI Prism 7300 system (Thermo Fisher Scientific). In this experiment, GAPDH was used as an internal reference gene, and all primer sequences are shown in Table S1.

## 3. Results

### 3.1. Identification of Core Differentially Expressed RRGs and Redox Patterns in PC

**Figure 1:**
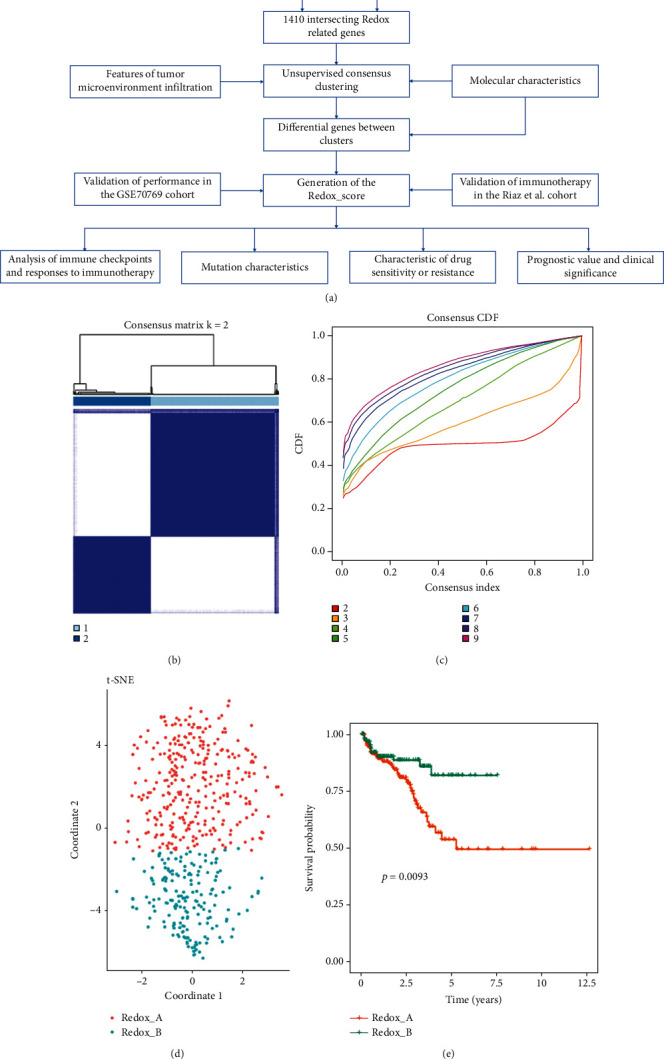
Identification of redox patterns in PC by unsupervised consensus clustering based on TCGA cohort. (a) The overall flow chart of this study. (b) Matrix heat map of *k*-means clustering based on 1410 differentially expressed RRGs. (c) CDF curve of *k*-means clustering. (d) The two-dimensional distribution of t-SNE at *k* = 2. (e) Kaplan-Meier survival curve of biochemical relapse (BCR) for PC patients in the TCGA cohort based on redox patterns.

Figure 1(a) shows the analysis flow chart of this study. After 4087 RRGs were obtained from the OMIM, NCBI, GeneCards, and GSEA-MSigDB databases, differential expression analysis was performed in TCGA-PRAD and GEO-PRAD cohorts. Then, we got 1410 overlapping RRGs from both cohorts. Next, we conducted unsupervised consensus clustering of PC samples in TCGA (*n* = 489) and GEO (*n* = 214) based on the 1410 RRGs. By calculating the cophenetic correlation coefficients of the two cohorts (delta area and CDF curve), *k* = 2 was chosen as the optimal cluster number (Redox_A and Redox_B, Figures 1(b) and 1(c), Figure S1). When *k* = 2, the boundary between the heat maps of the consistency matrix remains clear, indicating that the sample classification was robust. 305 patients in the TCGA cohort were assigned to Redox_A, and 184 were assigned to Redox_B. In the GEO cohort, 103 patients were assigned to Redox_A and 111 were assigned to Redox_B. In order to further verify the subclass assignment, we also performed t-SNE dimension reduction, and the results showed that the t-SNE distribution was consistent with the subclass (Figure 1(d), Figure S1D). Here, we defined the biochemical relapse (BCR) in PC patients as an end point event and compared outcomes between subclasses in the TCGA cohort. Kaplan–Meier analysis revealed that Redox_B had a significant survival advantage in BCR-free survival compared with Redox_A (*P* = 0.009, Figure 1(e)). These results suggested that these RRGs presented two subclasses with different survival advantages in PC, which required further analysis.

### 3.2. Molecular and Tumor Microenvironment Characteristics of Different Redox Patterns in PC

**Figure 2:**
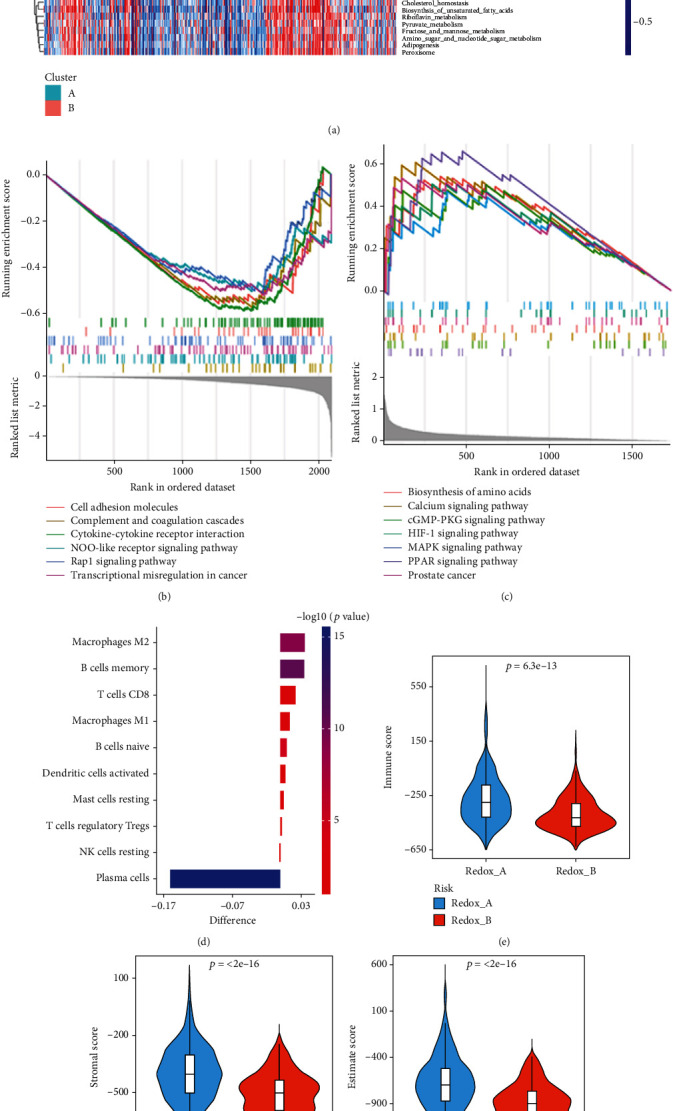
Molecular and tumor microenvironment characteristics of different PC subclasses. (a) Heat map of GSVA enrichment based on Hallmark and KEGG pathways from MSigDB in the redox patterns. (b) Heat map of GSEA enrichment of Redox_A pattern in PC. (c) Heat map of GSEA enrichment of Redox_B pattern in PC. (d) The distribution difference of immune infiltrating cells between redox patterns was analyzed by CIBERSORT algorithm. Differences > 0 indicated that immune cells were enriched in Redox_A pattern. The boxplot was the immune score (e), stromal score (f), ESTIMATE score (g), and tumor purity (h) of the redox patterns calculated by the ESTIMATE algorithm.

In order to understand the biological process of redox patterns in PC, GSVA enrichment and pathway difference analyses were performed, and the results were filtered according to ∣log_2_ FC | >0.15 and adjusted *P* < 0.05 (Figure 2(a)). The results showed that the Redox_A pattern was significantly enriched in carcinogenic activation signaling pathways, such as the JAK-STAT signaling pathway, TGF-*β* signaling pathway, ECM receptor interaction, and NOD-like receptor signaling pathway, which also partly explained why Redox_A has a shorter BCR-free survival time. The Redox_B pattern was mainly enriched in various energy metabolism pathways, such as arginine and proline metabolism, glutathione metabolism, tyrosine metabolism, fatty acid metabolism, and oxidative phosphorylation. Then, we further applied GSEA enrichment analysis to identify the enrichment pathways in each subclass. The results showed that the Redox_A pattern was significantly enriched in cell adhesion molecules, rap1 signaling pathway, NOD-like receptor signaling pathway, and transcriptional misregulation in cancer (Figure 2(b)), while the Redox_B pattern was significantly enriched in the biosynthesis of amino acids, calcium signaling pathway, HIF-1 signaling pathway, MAPK signaling pathway, cGMP-PKG signaling pathway, and PPAR signaling pathway (Figure 2(c)).

Next, the differences in immune-related characteristics among the subtypes of PC revealed by the above analysis results prompted us to further explore the infiltrating characteristics of the tumor microenvironment. We first used the CIBERSORT algorithm to evaluate the abundance of infiltrated immune cells in each sample of different redox patterns in PC, and the results were shown in Figure 2(d). Specifically, the infiltration of M2 macrophages (*P* = 9.5*e* − 10), memory B cells (*P* = 2.2*e* − 11), CD8 T cells (*P* = 0.007), M1 macrophages (*P* = 0.003), naive B cells (*P* = 1.4*e* − 5), activated dendritic cells (*P* = 0.029), resting mast cells (*P* = 0.049), T regulatory cells (Tregs) (*P* = 0.024), and resting NK cells (*P* = 0.014) was higher in Redox_A, while the infiltration of plasma cells (*P* = 4.0*e* − 16) was higher in Redox_B. Moreover, we further evaluated the tumor microenvironment of each sample in different PC subclasses by the ESTIMATE algorithm to determine their stromal score, immune score, ESTIMATE score, and tumor purity. The results show that in pattern Redox_A, immune score, stromal score, ESTIMATE score, and tumor purity were −274.825 ± 188.801 (Figure 2(e)), −400.188 ± 139.841 (Figure 2(f)), −675.013 ± 290.894 (Figure 2(g)), and 0.874 ± 0.021 (Figure 2(h)), respectively, while in pattern Redox_B, immune score, stromal score, ESTIMATE score, and tumor purity were −385.982 ± 122.966 (Figure 2(e)), −510.698 ± 111.203 (Figure 2(f)), −896.680 ± 199.951 (Figure 2(g)), and 0.890 ± 0.013 (Figure 2(h)), respectively. Compared with pattern Redox_B, the stromal cell and immune cell infiltration level in pattern Redox_A was higher, but the tumor purity was decreased.

Studies have shown that the number of CD4^+^CD25^+^Foxp3^+^ inhibitory regulatory T cells increases in peripheral blood of PC patients, and the ratio of CD4+/CD8^+^T cells is unbalanced, indicating that PC patients may be in a state of immunosuppression [19]. In addition, the previous analysis showed that M2 macrophages were associated with a higher Redox_score, which also supported this conclusion. These results suggest that there may be infiltration of immunosuppressed myeloid cells in PC. Therefore, we further analyzed the correlation between myeloid markers (ITGAM, OLR1, CD84, CD33, CD14, and VSIR) and the Redox_score. Figure S2A shows significant positive correlations between these myeloid marker molecules and between these molecules and the Redox_score. At the same time, it was found that the expression levels of these myeloid marker molecules in the high-risk group were significantly higher than those in the low-risk group (Figure S2B, C, D, E, F, G). Further Kaplan–Meier survival analysis showed that patients in the high-risk group had a poor prognosis in both the high and low expression groups of these molecules (Figure S2H, I, J, K, L, M).

### 3.3. Exploration Differential RRGs Associated with Redox Phenotype and Construction of a Redox_Score

**Figure 3:**
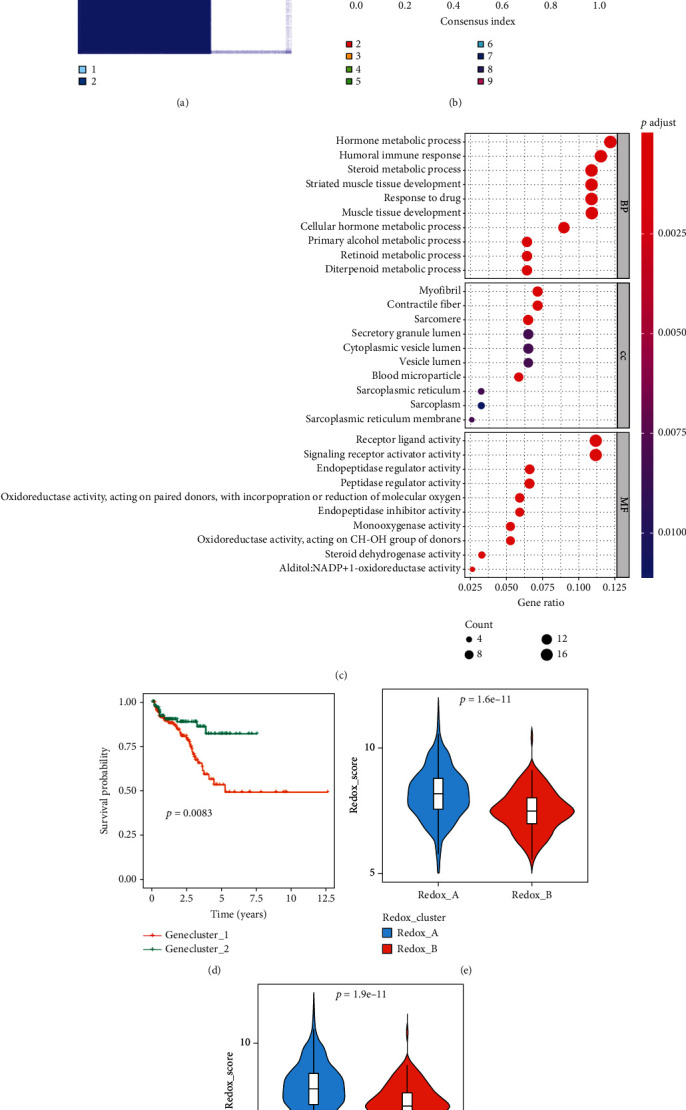
Identification of redox gene characteristic patterns in PC by unsupervised consensus clustering based on TCGA cohort. (a) Matrix heat map of *k*-means clustering based on 157 differentially expressed RRGs. (b) CDF curve of *k*-means clustering. (c) GO enrichment analysis of differentially expressed RRGs. (d) Kaplan-Meier survival curve of BCR for PC patients in the TCGA cohort based on gene characteristic patterns. The difference of Redox_score between redox patterns (e) and gene characteristic patterns (f) in TCGA cohort.

Although the RRG-based unsupervised consensus clustering classified PC patients into two redox phenotypes, the potential genetic changes and expression disturbances in these phenotypes were not clear. Based on these doubts, we further explored possible changes in redox-related transcriptional expression in these two PC patterns. Differential expression analysis of the two redox patterns was performed through the “Limma” R package according to the screening criteria of ∣log_2_ FC | >1 and *P* < 0.05; a total of 157 differentially expressed RRGs were obtained. Based on these RRGs, we conducted unsupervised consensus clustering analysis and finally selected *k* = 2 as the optimal cluster number after comprehensive consideration and divided PC patients into two different redox gene characteristic subgroups (Genecluster_1 and Genecluster_2) (Figures 3(a) and 3(b)). Gene ontology analysis revealed that these RRGs were also mainly enriched in various metabolic and carcinogenic biological processes, indicating that these RRGs could be used as characteristics of redox-related genes (Figure 3(c)). We further conducted Kaplan–Meier survival analysis, and the results showed that the prognosis of PC patients in the two gene patterns was significantly different; specifically, Genecluster_2 had a significant survival advantage in BCR-free survival (*P* = 0.008, Figure 3(d)).

Considering the heterogeneity and complexity of redox function in the tumor, we further screened the most prognostic redox characteristic RRGs to construct a score model to quantify the PC patients. We first performed univariate Cox regression analysis of these 157 RRGs and screened 46 prognostic RRGs. LASSO regression analysis was performed for these RRGs based on the “glmnet” R package, and the 11 most prognostic RRGs were identified. The trajectory changes of the 46 independent variable coefficients and the results of cross-validation are shown in Figure S3A, B. Subsequently, multiple stepwise Cox regression analysis was performed for these 11 RRGs and the optimal combinations were selected according to AIC (Figure S3C). Finally, 6 RRGs were obtained and a redox score (Redox_score) was constructed based on the following formula: Redox_score = (0.1713 × ExpAKR1C3) + (0.3673 × ExpCOL1A1) + (0.1393 × ExpCYP3A4) + (0.2434 × ExpMYBL2) + (0.0958 × ExpRALYL) + (−0.3018 × ExpSCN4A). We found that Redox_A had a higher Redox_score than Redox_B (*P* = 1.6*e* − 11, Figure 3(e)). Similarly, Genecluster_1 had a higher Redox_score than Genecluster_2 (*P* = 1.9*e* − 11, Figure 3(f)).

### 3.4. Evaluation of Redox_Score Performance

**Figure 4:**
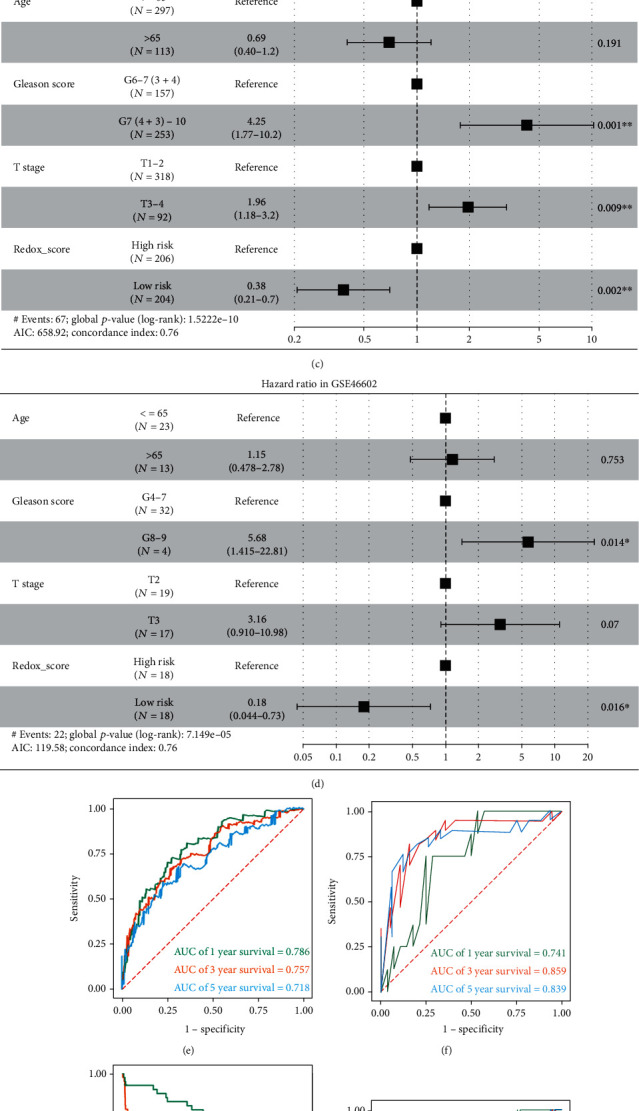
Evaluation of Redox_score performance. (a) Kaplan-Meier survival curve of BCR between low- and high-risk groups in the TCGA cohort. (b) Kaplan-Meier survival curve of BCR between low- and high-risk groups in the GSE46602 cohort. (c) Multivariate Cox regression analysis of age, Gleason score, stage, and Redox_score were included in the TCGA cohort. (d) Multivariate Cox regression analysis of age, Gleason score, stage, and Redox_score were included in the GSE46602 cohort. (e) Redox_score predicted AUC values at different time points in the TCGA cohort. (f) Redox_score predicted AUC values at different time points in the GSE46602 cohort. (g) Kaplan-Meier survival curve of BCR between low- and high-risk groups in the GSE70769 cohort. (h) Redox_score predicted AUC values at different time points in the GSE70769 cohort.

We grouped PC patients in the TCGA cohort (low-risk group and high-risk group) according to the calculated median Redox_score. Survival analysis revealed that the low-risk group had a significant survival advantage in BCR-free survival (*P* = 4.702*e* − 07, Figure 4(a)). The predicted area under the receiver operating characteristic (ROC) curve of Redox_score was 0.786, 0.757, and 0.718 at 1, 3, and 5 years, respectively (Figure 4(e)). The results of the Kaplan–Meier survival analysis and ROC analysis based on the GSE46602 cohort were consistent with the above (Figures 4(b) and 4(f)). To investigate whether Redox_score could independently predict patient outcomes, we included common clinical characteristics (including age, stage, Gleason score, and Redox_score) for multivariate Cox analysis. The results showed that the Redox_score was an independent and reliable prognostic factor for the prognosis of PC patients (HR = 0.380, 95% CI 0.210–0.700, *P* = 0.002, Figure 4(c)). We further used the GSE46602 cohort to verify the reliability of the Redox_score (HR = 0.180, 95% CI 0.044–0.730, *P* = 0.016, Figure 4(d)). Additionally, we also used the GSE70769 cohort to verify the predictive performance of the Redox_score, which was consistent with the above findings (Figures 4(g) and 4(h)). These results showed that the Redox_score had great predictive potential.

### 3.5. Exploration of the Redox_Score's Clinical Relevance and miRNA-RRG Regulatory Networks

**Figure 5:**
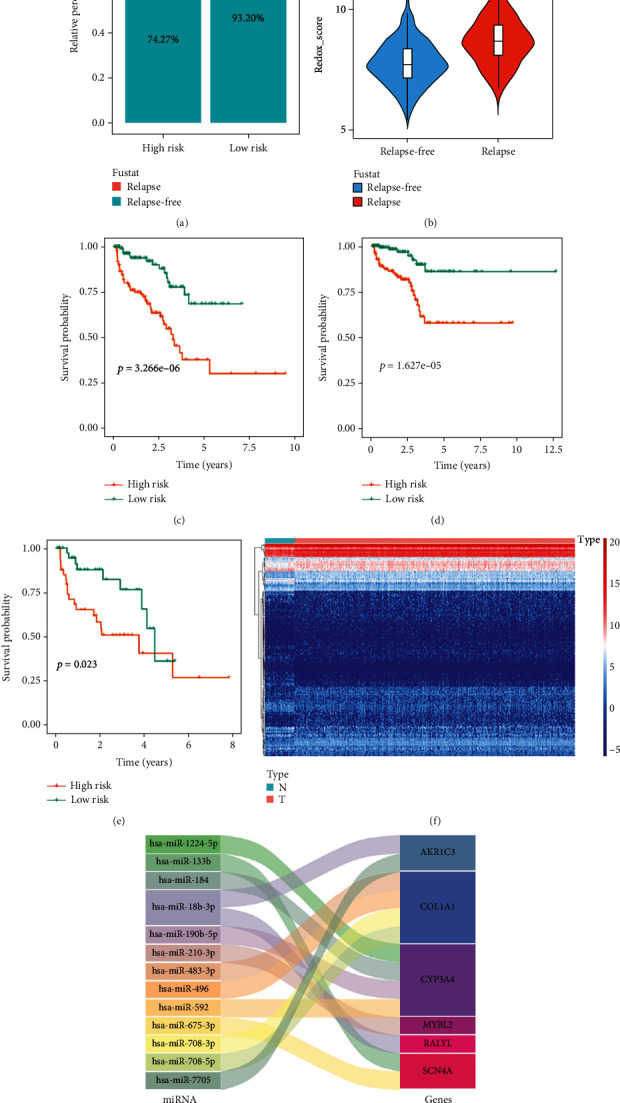
Exploration of the Redox_score's clinical relevance and miRNA-RRG regulatory networks. (a) Differences in BCR between low- and high-risk groups in PC patients. (b) Differences in Redox_score between patients with and without BCR. (c) Kaplan-Meier survival curve of BCR between low- and high-risk group G7(4+3)-10 patients in the TCGA cohort. (d) Kaplan-Meier survival curve of BCR between low- and high-risk group T stage 1-2 patients in the TCGA cohort. (e) Kaplan-Meier survival curve of BCR between low- and high-risk group T stage 3-4 patients in the TCGA cohort. (f) Expression heat map of differentially expressed miRNAs. N represents the normal group, and T represents the tumor group. (g) Sankey plot of differentially expressed miRNAs and prognostic RRG regulatory networks.

Next, we first evaluated the relationship between Redox_score and BCR. As shown in Figure 5(a), patients in the high-risk group had a higher rate of BCR than patients in the low-risk group (25.73% vs. 6.80%, *P* < 0.001). Similarly, the higher the BCR rate, the higher the Redox_score (*P* = 1.9*e* − 11, Figure 5(b)). Then, we stratified PC patients in the TCGA cohort with the Gleason score and T stage, and the results showed that low-risk patients in each stratification had a higher survival advantage (Figures 5(c)–5(e)).

miRNAs are a class of important regulatory factors that significantly affect the genesis and progression of tumors by regulating the entire cell signaling network [20, 21]. Meanwhile, mRNAs also have a great importance in maintaining ROS homeostasis, and many studies focused on the regulatory interaction between miRNA and ROS [22]. For example, miR-21 can mediate ROS production by enhancing KRAS and epidermal growth factor receptor signaling, thereby promoting tumor development [23, 24]. Therefore, this study deserved further attention on the relationship between miRNAs and prognostic RRGs and to reveal the regulatory network of miRNAs-RRGs. We obtained miRNA expression data from the TCGA cohort, including 52 normal samples and 499 PC samples. A total of 76 downregulated miRNAs and 118 upregulated miRNAs were obtained after differential analysis.  Figure 5(f) showed the heat map of differentially expressed miRNAs. Subsequently, we conducted the coexpression analysis of these 6 prognostic RRGs (the most prognostic redox characteristic RRGs) and these differential miRNAs and finally obtained 14 pairs of miRNA-RRG regulatory networks (Figure 5(g)). Here, all miRNAs were positively regulated corresponding RRGs. The specific regulatory relationship between these miRNAs and prognostic RRGs is shown in Table S2. Moreover, we further analyzed that the miRNAs related to the Redox_score correlated with Redox_A and Redox_B by coexpression analysis according to suggestion. According to the criteria of cor > 0.3 and *P* < 0.001, we found 11 miRNAs related to the Redox_score correlated with Redox_A. And according to the criteria of cor > 0.25 and *P* < 0.001, we found 15 miRNAs related to the Redox_score correlated with Redox_B (Table S3).

### 3.6. Correlation of the Redox_Score with Mutations

**Figure 6:**
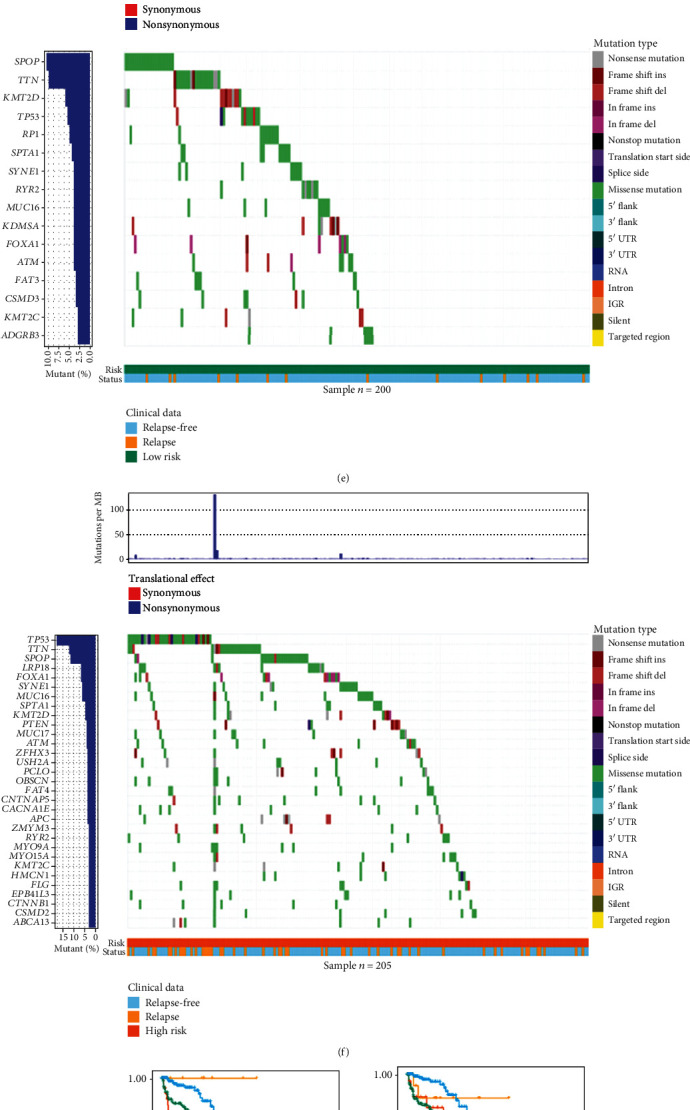
Correlation of the Redox_score with mutations. (a) Differences in TMB between low- and high-risk groups in PC patients. (b) Correlation analysis between Redox_score and TMB. (c) Kaplan-Meier survival curve of BCR between low- and high-TMB groups in the TCGA cohort. (d) Kaplan-Meier survival curve of BCR among four groups stratified by the Redox_score and TMB. (e) Mutation waterfall plot of patients in the low-risk group. (f) Mutation waterfall plot of patients in the high-risk group. Kaplan-Meier survival curve of BCR among four groups stratified by the Redox_score and TP53 (g), TTN (h), and SPOP (i).

Tumor genomic patterns have been shown to be associated with antitumor immunity. The accumulation of somatic mutations is one of the main causes of tumorigenesis [25]. TMB is also considered a biomarker for predicting tumor behavior and immune response [26]. Higher TMB has been reported to be associated with better prognosis in patients with melanoma and non-small cell lung cancer [27]. In order to investigate whether there were differences in somatic mutations in Redox_score and to observe the mutation patterns between Redox_score, we analyzed the data of somatic mutations in the TCGA cohort. The results showed that TMB levels were higher in the high-risk group (*P* = 2.1*e* − 08, Figure 6(a)), and there was a significant positive correlation between the Redox_score and TMB (*R* = 0.31, *P* = 8.6*e* − 11, Figure 6(b)), indicating that TMB increased with the increase of the Redox_score. We further investigated whether TMB was associated with survival advantage, and the analysis indicated that low TMB had a significant survival advantage in BCR-free survival (*P* = 0.005, Figure 6(c)). On this basis, we investigated whether the Redox_score still had an impact on the prognosis of patients when the level of TMB was considered. The results showed that high TMB and low-risk patients had a significant survival advantage in BCR-free survival compared with the high TMB and high-risk patients, and the low TMB and low-risk patients had also a significant survival advantage in BCR-free survival compared with the low TMB and high-risk patients (*P* < 0.001, Figure 6(d)). Next, we showed the driver genes that were mutated in at least 3% of the samples in the low- and high-risk patients. The results indicated that there were more mutated driver genes in the high-risk patients (Figures 6(e) and 6(f)). We selected three driver genes (TP53, TTN, and SPOP) with high mutation rates and explored whether the Redox_score still had an impact on the prognosis of patients when the expression of driver genes was considered. The results indicated that the TP53 mutation and low-risk patients had a significant survival advantage in BCR-free survival compared with the TP53 mutation and high-risk patients, and the TP53 wild and low-risk patients had also a significant survival advantage in BCR-free survival compared with the TP53 wild and high-risk patients (*P* < 0.001, Figure 6(g)). Consistent results were observed in other driver genes (Figures 6(h) and 6(i)). Considering that PTEN and AR mutations have important clinical significance for PC patients, we further analyzed whether Redox_score still has an impact on the prognosis of patients when considering PTEN and AR mutations. We analyzed PTEN in the TCGA-PRAD dataset and AR in the metastatic PC dataset (Abida et al. PNAS 2019, cBioPortal, https://www.cbioportal.org/). The results of Kaplan–Meier analysis showed that the prognosis of high-risk patients was poor in both the AR (or PTEN) mutation group and the AR (or PTEN) wild group (Figure S4).

### 3.7. Benefit of Redox_Score in Predicting Immunotherapy Reactivity

**Figure 7:**
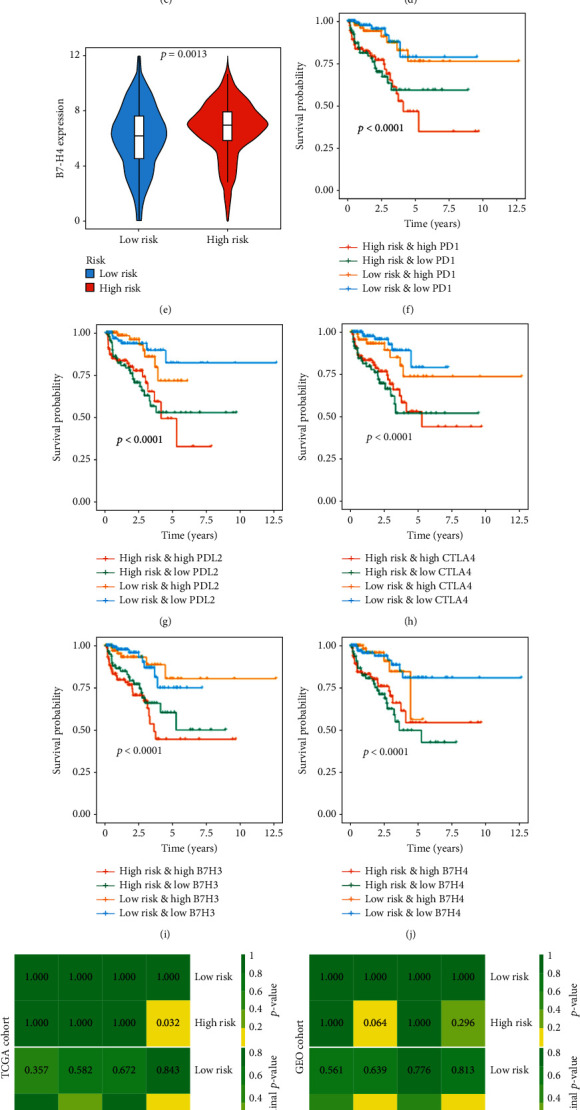
Benefit of Redox_score in predicting immunotherapy reactivity. Differences in expression of PD-1 (a), PD-L2 (b), CTLA4 (c), B7-H3 (d), and B7-H4 (e) between low- and high-risk groups. Kaplan-Meier survival curve of BCR among four groups stratified by the Redox_score and PD-1 (f), PD-L2 (g), CTLA4 (h), B7-H3 (i), and B7-H4 (j). (k) Similarity of gene expression profiles between redox patterns and ICI-treated melanoma patients in the TCGA cohort. (l) Similarity of gene expression profiles between redox patterns and ICI-treated melanoma patients in the GEO cohort. (m) Kaplan-Meier survival curve of BCR between low- and high-risk groups in the Riaz et al. cohort.

Immunotherapy targeting ICI genes has been a major breakthrough in antitumor therapy in recent years [28]. In order to further study the complex interaction between ICI genes and Redox_score, we first explored the expression of these genes (PD-1, PD-L2, CTLA-4, B7-H3, and B7-H4) in different patient groups of patients under stratification of the Redox_score. The results indicated that compared with the low-risk patients, the expression levels of PD-1 (*P* = 0.014, Figure 7(a)), PD-L2 (*P* = 0.018, Figure 7(b)), CTLA-4 (*P* = 5*e* − 05, Figure 7(c)), B7-H3 (*P* = 1.9*e* − 05, Figure 7(d)), and B7-H4 (*P* = 0.001, Figure 7(e)) in the high-risk patients were significantly upregulated, which was similar to the result of Sun et al.'s [29] study that the expression level of the ICI gene was negatively correlated with the prognosis of patients. We also investigated whether the Redox_score still had an impact on the prognosis of patients when the expression level of the ICI gene was considered. The results showed that the low-risk and high PD-1 expression patients had a significant survival advantage in BCR-free survival compared with the high-risk and high PD-1 expression patients, and the low-risk and low PD-1 expression patients had also a significant survival advantage in BCR-free survival compared with the high-risk and low PD-1 expression patients (*P* < 0.001, Figure 7(f)). Interestingly, when we stratified the Redox_score based on the level of expression of the ICI gene, the results showed that the low-risk (or high-risk) and low PD-1 expression patients had no significant survival advantage in BCR-free survival compared with the low-risk (or high-risk) and high PD-1 expression patients. Similar results were found for other genes (Figures 7(g)–7(j)). These results suggested that the Redox_score may be a potential marker for predicting response to immunotherapy in patients with PC. By SubMap analysis, we further compared the expression data of Redox_scores from the TCGA and GEO cohorts, with another available dataset of 47 melanoma patients receiving PD-1 or CTLA-4 immunotherapy. Both cohorts showed a significant correlation between the high-risk patients and the PD-1 response (*P* = 0.004 and *P* = 0.037, Figures 7(k) and 7(l)), suggesting that patients with a higher Redox_score were more responsive to PD-1 immunotherapy. Based on these results, we obtained an immunotherapy cohort of patients undergoing anti-PD-1 therapy for metastatic melanoma to further assess whether the Redox_score could predict patient response to ICI. The results indicated that the low-risk patients had also a significant survival advantage in BCR-free survival (*P* = 0.012, Figure 7(m)). These results indicated that the Redox_score was associated with response to immunotherapy and could further predict PC patient outcomes.

### 3.8. Correlation Analysis between Redox_Score and Drug Sensitivity

**Figure 8:**
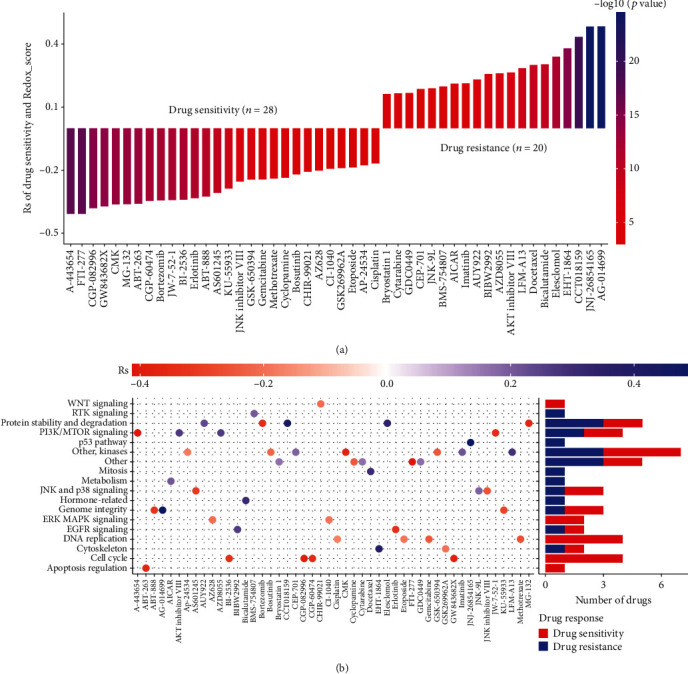
Correlation analysis between Redox_score and drug sensitivity. (a) Pearson correlation analysis was used to evaluate the correlation between Redox_score and drug sensitivity and drug resistance. (b) Related signaling pathways of drug-targeted genes associated with Redox_score.

To explore the effect of the Redox_score on drug sensitivity of tumor cells, we further evaluated the correlation between the Redox_score and the drug response in the GDSC database. We performed the Pearson correlation analysis between the Redox_score and the drug response of cancer cell lines, and based on ∣Rs | >0.15 and *P* < 0.05 screening criteria, 48 significant correlation pairs were identified (Figure 8(a)). Of these, 28 correlation pairs showed drug sensitivity associated with the Redox_score. For example, A-443654 (Rs = −0.41, *P* = 6.42*e* − 18), FTI-277 (Rs = −0.41, *P* = 8.03*e* − 18), CGP-082996 (Rs = −0.38, *P* = 1.79*e* − 15), and GW843682X (Rs = −0.37, *P* = 7.76*e* − 15), and 20 correlation pairs showed drug resistance associated with the Redox_score. For example, AG-014699 (Rs = 0.48, *P* = 2.76*e* − 25), JNJ-26854165 (Rs = 0.48, *P* = 2.72*e* − 25), CCT018159 (Rs = 0.43, *P* = 2.60*e* − 20), and EHT-1864 (Rs = 0.38, *P* = 1.97*e* − 15). We further analyzed the related signaling pathways of the drug-targeted genes mentioned above. The results indicated that the Redox_score was related to drug sensitivity to targeted apoptosis regulation, cell cycle, DNA replication, and ERK MAPK signaling pathways, while the Redox_score was related to drug resistance to targeted hormone-related, p53, and PI3K/MTOR signaling pathways (Figure 8(b)), indicating that higher Redox_score patients may be more effective for drugs targeting apoptosis or the cycle pathway, while lower Redox_score patients may be more effective for drugs targeting hormone-related or p53 pathway. These results indicated that the Redox_score was related to the sensitivity of tumor cells to drugs and could be used as a potential biomarker.

### 3.9. RT-qPCR Verification

**Figure 9:**
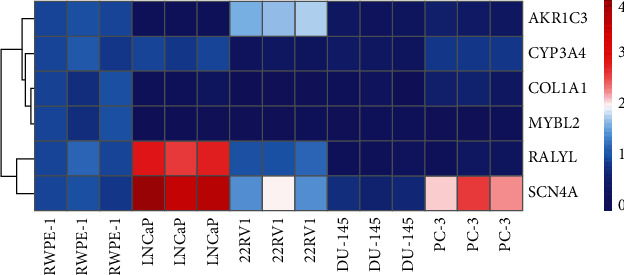
The expression heat map of prognostic redox characteristic RRGs in the normal prostate epithelial cells (RWPE-1), hormone-dependent prostate cancer cells (LNCaP), and hormone-resistant prostate cancer cells (22RV1, DU-145, and PC-3).

To further evaluate the reliability of the Redox_score, we detected the actual expression levels of these six redox characteristic genes in normal prostate epithelial cells (RWPE-1), hormone-dependent PC cells (LNCaP), and hormone-resistant PC cells (22RV1, DU-145, and PC-3) by RT-qPCR. The analysis results of the experiment are shown in Figure 9. AKR1C3 expression was significantly downregulated in LNCaP, DU-145, and PC-3 cells, while significantly upregulated in 22RV1 cells compared with RWPE-1 cells. Compared with RWPE-1 cells, CYP3A4 expression was significantly downregulated in 22RV1 and DU-145 cells, while there was no significant difference in LNCaP and PC-3 cells. COL1A1 and MYBL2 were significantly downregulated in LNCaP, 22RV1, DU-145, and PC-3 cells compared with RWPE-1 cells. Compared with RWPE-1 cells, the RALYL expression was significantly upregulated in LNCaP cells and downregulated in DU-145 and PC-3 cells, while there was no significant difference in the RALYL expression in 22RV1 cells. Compared with RWPE-1 cells, SCN4A was significantly upregulated in LNCaP, 22RV1 and PC-3 cells, while downregulated in DU-145 cells.

## 4. Discussion

Imbalance of redox homeostasis has been shown to be closely related to cancer genesis, proliferation, invasion, and vascularization [30, 31]. In addition, ROS components produced by a variety of inflammatory cells located in the tumor microenvironment, such as superoxide and hydrogen peroxide, can further affect the function of cancer cells and adjacent immune cells [30]. Although numerous studies have revealed the different roles of redox in numerous cancer-related processes, most current studies have only explored the function of a single redox gene; the overall characteristics of cancer mediated by comprehensive redox genes, as well as their relationships and functions in cancer, have not yet been fully understood. Therefore, a comprehensive and effective analysis of redox modification patterns and characteristics in the PC tumor will contribute to a deeper understanding of the role of redox in the PC tumor and its interrelationship and promote more effective and precise treatment strategies. Here, we first identified two different redox modification patterns based on 1410 differentially expressed RRGs in TCGA and GEO cohorts. We analyzed the characteristics of the molecular and the tumor immune microenvironment of these two patterns. Next, we further identified two redox characteristic gene patterns through redox characteristic genes. Finally, we constructed a scoring system and assessed its benefit in predicting patient outcomes, responses to immunotherapy, and sensitivity or resistance to drug responses.

In this study, we identified two redox patterns based on differentially expressed RRGs. In these two patterns, the molecular characteristics of pattern A were significantly enriched in carcinogenic activation signaling pathways, for example, the JAK-STAT signaling pathway, TGF-*β* signaling pathway, ECM receptor interaction, and NOD-like receptor signaling pathway, while pattern B was mainly enriched in metabolic- and redox-related pathways, for example, arginine and proline metabolism, glutathione metabolism, tyrosine metabolism, fatty acid metabolism, peroxisome, and oxidative phosphorylation. Activation of the TGF-*β* signaling pathway regulates gene expression in a variety of cell biological processes, including cell proliferation, apoptosis, invasion, epithelial-mesenchymal transformation, and immune regulation [32]. Moreover, studies have shown that TGF-*β* is closely related to the invasion and metastasis of advanced cancer cells [33]. In the case of PC, TGF-*β*-regulated vimentin levels were significantly associated with patients' BCR, with TGF-*β*3 ligand being more able to control the metastatic behavior of cancer cells [34, 35]. Activation of the JAK-STAT signaling pathway is also a common event in multiple stages of carcinogenesis in PC [36]. Not surprisingly, patients with pattern A fared worse than those with pattern B. Pattern B enrichment in metabolic and redox signals suggested that these patients may benefit from metabolic therapy. Metabolic therapy, which targets certain metabolic processes, offers alternative therapies for these patients. Given the complex interrelationship between redox homeostasis and metabolic pathways in cancer, multiple studies have focused on the treatment of cancer by targeting ROS with metabolic regulators. For instance, several studies have revealed that orlistat, as an antitumor drug, inhibits tumor growth in a variety of cancers, including prostate cancer, by inhibiting fatty acid synthase [37]. Biguanides (metformin and metformin) increase the AMP/ATP ratio mainly by inhibiting mitochondrial respiratory chain complex I, thus activating AMPK, further inducing catabolism process, increasing ATP level, reducing protein and lipid synthesis, and ultimately inhibiting tumor growth [38, 39]. This would provide new insights into metabolic therapy as an alternative therapy.

Next, we identified differentially expressed genes from the two patterns, which were significantly enriched in various metabolic pathways and immunoregulatory biological processes, and were considered to be gene characteristics associated with redox phenotypes. We also identified two gene patterns based on these redox signature genes and further constructed a scoring system (Redox_score) based on the redox signature genes most associated with prognosis to more accurately guide the treatment strategies of individual patients. We found that the A pattern, characterized by stromal and carcinogenic activation pathways, had a higher Redox_score, while the B pattern, characterized by metabolic and redox pathways, had a lower Redox_score. Further analysis found that the Redox_score independently predicted the prognosis of patients with PC in both the TCGA and GEO cohorts, suggesting that the Redox_score had a high predictive potential in patients with PC.

The continuous accumulation of somatic mutations is one of the important causes of tumorigenesis and contributes to the production of new antigens [24]. Therefore, the evaluation of mutation-driving genes in human tumors is an important basis for cancer diagnosis and treatment formulation. Here, a significant positive correlation was found between Redox_score and TMB. In addition, we found that SPOP was the most mutated driver gene in the low-risk patients, while TP53 was the most mutated driver gene in the high-risk patients. Several studies have revealed that SPOP inhibited the progression of PC by promoting the degradation of various oncoproteins, such as androgen receptor [40], steroid receptor coactivator 3 [41], and Myc [42]. However, SPOP mutation rates have been reported to reach 6-15% in local and advanced PC [43], so SPOP mutations have been identified as an early event in the occurrence and progression of PC, partly due to genetic instability [44]. TP53 exerted the tumor inhibitory effect by regulating signaling pathways such as genomic stabilization and cell cycle arrest [45]. TP53 has a higher mutation frequency in patients with PC, affecting 50% of patients with metastatic PC [46]. Multiple studies have shown that TP53 levels have prognostic significance in castration-resistant PC and serve as a biomarker of adverse responses to novel hormone therapy [47].

The latest EAU guidelines indicated the currently approved ICI in PC target molecules CTLA4, PD-1, and PD-L1. Anti-CTLA-4 or anti-PD-1 monotherapy or combination immunotherapy was currently being rigorously tested in PC [48]. A phase II trial of 258 PC patients treated with pembrolizumab showed an objective response rate of about 4%, but these responses were long-lasting [49]. In our study, we also explored the expression relationship between common ICI genes (PD-1, PD-L2, CTLA4, B7-H3, and B7-H4) and the Redox_score, and we found that compared with the low-risk patients, the expression levels of ICI genes in the high-risk patients were significantly upregulated. Immune checkpoint refers to a class of inhibitory or irritating molecules expressed mainly on tumor cells, antigen presenting cells, or immune cells. Immune checkpoint molecules expressed in antigen-presenting cells or immune cells mainly mediate the processes of the adaptive and innate immune systems. The immune checkpoint molecules expressed in different types of tumors play important roles in tumor cell biology, such as inducing epithelial-mesenchymal transformation, promoting tumor initiation, and promoting tumor metastasis, antiapoptosis, and antitumor drug resistance [50, 51]. Our results were consistent with previous findings that the expression level of the ICI gene was negatively correlated with the prognosis of patients [29, 50]. Moreover, SubMap analysis indicated that the high-risk patients were more responsive to ICI than the low-risk patients, which was consistent with the above results. We also further determined the potential value of the Redox_score in predicting immunotherapy reactivity by analyzing the immunotherapy cohort of patients receiving PD-1 treatment for melanoma. We believed that our Redox_score will be useful in assessing the benefit of patients receiving anti-CTLA-4 or anti-PD-1 immunotherapy. Therefore, accessible datasets of PC patients receiving immunotherapy were needed to further validate these results. Finally, the correlation between Redox_score and drug sensitivity and drug resistance was also analyzed. We found that the drugs related to Redox_score-high mostly targeted apoptosis regulation, cell cycle, DNA replication, and ERK MAPK signaling pathways, while the drugs related to Redox_score-low mostly targeted hormone-related, p53, and PI3K/MTOR signaling pathways.

Overall, although we have analyzed the overall redox modification profile in PC and developed a scoring system with prognostic potential, there are some limitations to this study. First, our study was based on retrospective datasets to determine redox modification patterns and Redox_score, and prospective cohorts are needed to validate our results. Secondly, the specific biological functions and molecular mechanisms of RRGs affecting the prognosis of PC are still unclear, and further analysis is needed through experiments. Thirdly, the Redox_score needs to be further validated in open-access expression data of cancer patients receiving antioxidant therapy. Finally, we used available immunotherapy cohorts for other tumor (melanoma) to validate the role of the Redox_score, which needs further validation with available PC immunotherapy cohorts.

## 5. Conclusions

In our study, we systematically and comprehensively assessed the redox modification patterns of RRGs in PC, revealing their molecular mechanisms and immune microenvironment characteristics in PC. We also constructed a scoring system (Redox_score) and identified its upstream regulatory network, prognostic value, benefit in predicting immunotherapeutic response, and drug sensitivity relationships in PC, which will help to develop individualized treatment strategies for PC patients.

## Data Availability

The data and materials can be obtained by contacting the corresponding author.

## Abbreviations

PC: Prostate cancer
ROS: Reactive oxygen species
RNS: Reactive nitrogen
TCGA: The Cancer Genome Atlas
GEO: Gene Expression Omnibus
RRGs: Redox-related genes
GSVA: Gene set variation analysis
CIBERSORT: Cell-type identification by estimating relative subsets of RNA transcripts
FC: Fold change
LASSO: Least absolute shrinkage and selection operator
TMB: Tumor mutation burden
ICI: Immune checkpoint inhibitor
GDSC: Genomics of Drug Sensitivity in Cancer
BCR: Biochemical relapse
ROC: Receiver operating characteristic.

## Supplementary Materials

Supplementary Materials Supplemental Figure S1: identification of redox patterns in PC by unsupervised consensus clustering based on GEO cohort. Supplemental Figure S2: correlation analysis between myeloid marker molecules and the Redox_score. Supplemental Figure S3: identification of prognosis-related RRGs. Supplemental Figure S4: correlation of the Redox_score with PTEN and AR mutations. Supplemental Table S1: the sequences of primer RNA. Supplemental Table S2: miRNA and RRG regulatory networks. Supplemental Table S3: miRNAs related to which Redox_score they correlated with Redox_A and Redox_B.
