# Supplementary material for: Exploration of Redox-Related Molecular Patterns and the Redox Score for Prostate Cancer
Source: Oxid Med Cell Longev. 2021 Nov 11;2021:4548594. doi: 10.1155/2021/4548594 (PMC8601839; doi:10.1155/2021/4548594)
Supplement: Supplementary Materials — Supplemental Figure S1: identification of redox patterns in PC by unsupervised consensus clustering based on GEO cohort. Supplemental Figure S2: correlation analysis between myeloid marker molecules and the Redox_score. Supplemental Figure S3: identification of prognosis-related RRGs. Supplemental Figure S4: correlation of the Redox_score with PTEN and AR mutations. Supplemental Table S1: the sequences of primer RNA. Supplemental Table S2: miRNA and RRG regulatory networks. Supplemental Table S3: miRNAs related to which Redox_score they correlated with Redox_A and Redox_B. [file 4548594.f1.docx]

**Exploration of redox related molecular patterns and the redox score for prostate cancer**


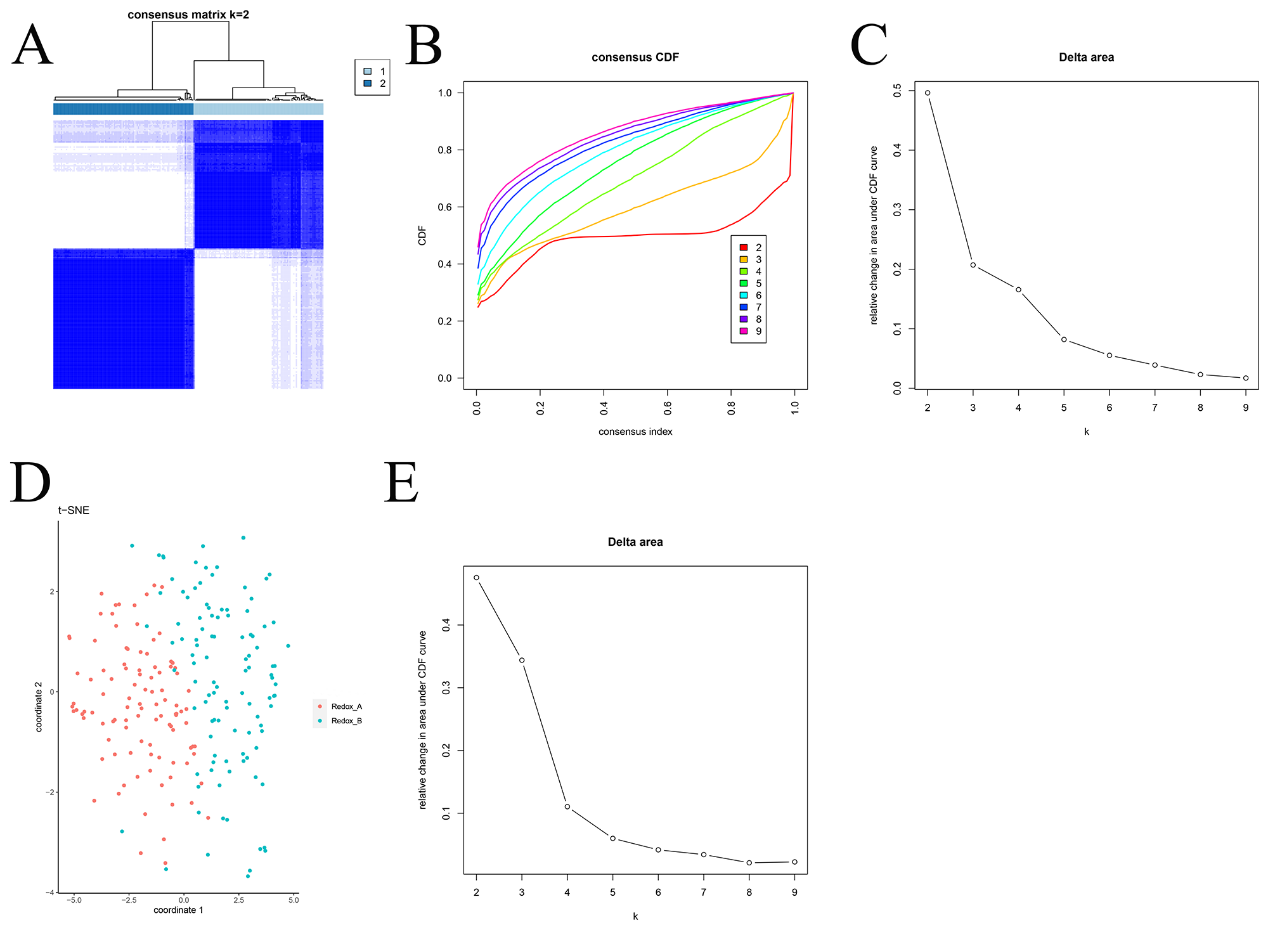


Supplemental Figure S1. Identification of redox patterns in PC by unsupervised consensus clustering based on GEO cohort.

(A) Matrix heatmap of k-means clustering based on 1410 differentially expressed RRGs. (B) CDF curve of k-means clustering. (C) Delta area of k-means clustering in the GEO cohort. (D) The two-dimensional distribution of t-SNE at k=2. (E) Delta area of k-means clustering in the TCGA cohort.


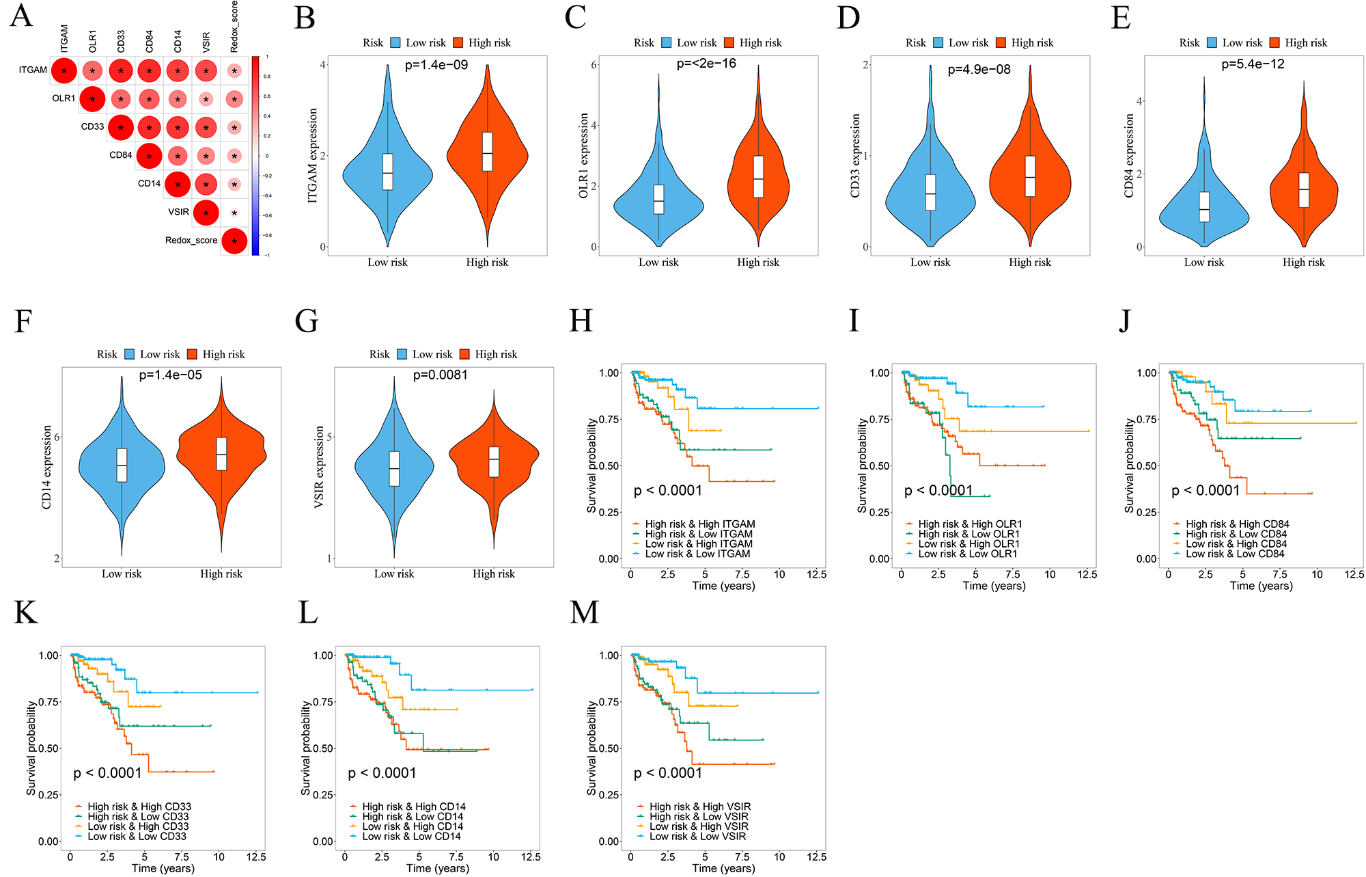


Supplemental Figure S2. Correlation analysis between myeloid marker molecules and the Redox_score.

(A) Correlation between myeloid marker molecules and the Redox_score in PC. Differences in expression of ITGAM (B), OLR1 (C), CD33 (D), CD84 (E), CD14 (F), and VSIR (G) between low- and high-risk groups. Kaplan-Meier survival curve of BCR among four groups stratified by the Redox_score and ITGAM (H), OLR1 (I), CD84 (J), CD33 (K), CD14 (L), and VSIR (M).


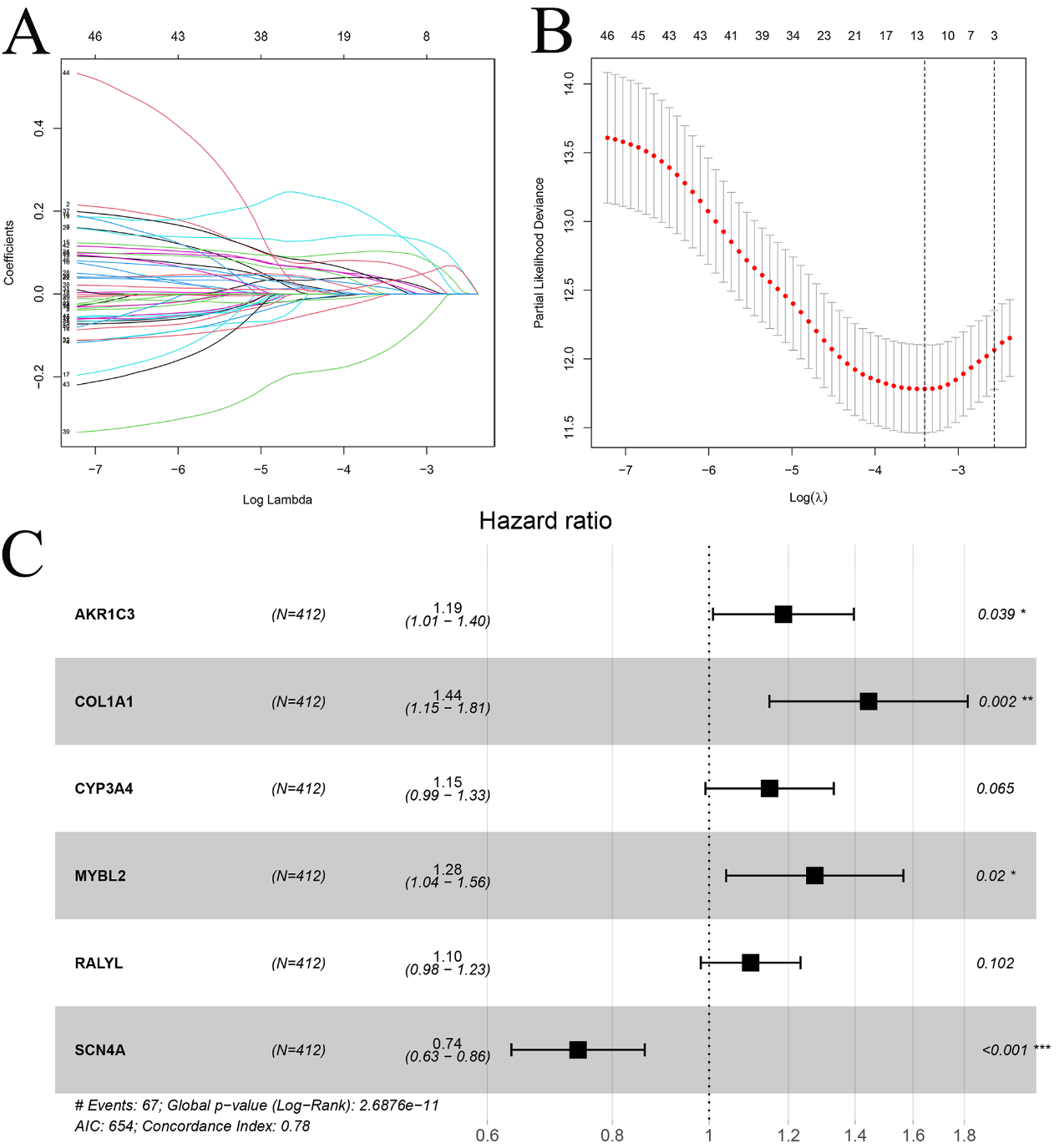


Supplemental Figure S3. Identification of prognosis related RRGs.

(A) Trajectories of model coefficients. (B) Cross validation fitting and performance

evaluation of the model. (C) Multivariate Cox regression analysis of 6 RRGs.


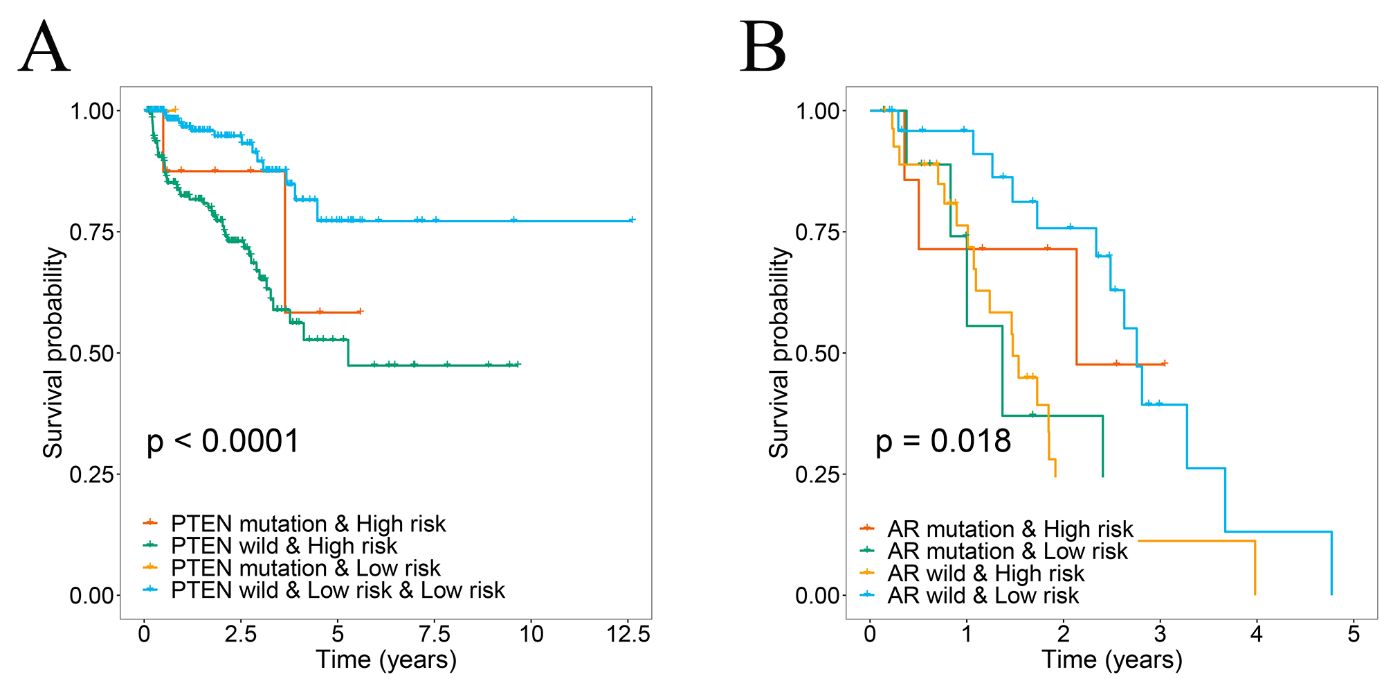


Supplemental Figure S4. Correlation of the Redox_score with PTEN and AR mutations.

(A) PTEN. (B) AR.

Table S1. The sequences of primer RNA.

| Name | Primer | Sequence | Size |
| --- | --- | --- | --- |
| Human GAPDH | Forward | 5‘- GAGAAGGCTGGGGCTCATTT-3’ | 231bp |
|  | Reverse | 5‘- AGTGATGGCATGGACTGTGG-3’ |  |
| Human AKR1C3 | Forward | 5‘- TCACTTCATGCCTGTCCTGG-3’ | 104bp |
|  | Reverse | 5‘- GGCGGAACCCAGCTTCTATT-3’ |  |
| Human COL1A1 | Forward | 5‘- AGTGGTTTGGATGGTGCCAA-3’ | 170bp |
|  | Reverse | 5‘- GCACCATCATTTCCACGAGC-3’ |  |
| Human CYP3A4 | Forward | 5‘- AAGGGATGGCACCGTAAGTG-3’ | 174bp |
|  | Reverse | 5‘- GCATGTACAGAATCCCCGGT-3’ |  |
| Human MYBL2 | Forward | 5‘- CCATGAGGAGAACCGCACTG-3’ | 160bp |
|  | Reverse | 5‘- GTGCTTGGCAATCAGTGTCC-3’ |  |
| Human RALYL | Forward | 5‘- AGGGAACAGCAGAGGCAAAG-3’ | 192bp |
|  | Reverse | 5‘- GCTGGTCTGTGTTTTGCCAG-3’ |  |
| Human SCN4A | Forward | 5‘- GTCTTTGCGCTGGTAGGACT -3’ | 188bp |
|  | Reverse | 5‘- GTCTTTGCGCTGGTAGGACT -3’ |  |

Table S2. MiRNAs and RRGs regulatory networks.

| miRNAs | RRGs | Coefficient | P value | Regulation |
| --- | --- | --- | --- | --- |
| hsa-miR-18b-3p | AKR1C3 | 0.349 | 4.29E-13 | Positive |
| hsa-miR-7705 | AKR1C3 | 0.455 | 3.62E-22 | Positive |
| hsa-miR-483-3p | COL1A1 | 0.303 | 4.81E-10 | Positive |
| hsa-miR-496 | COL1A1 | 0.311 | 1.54E-10 | Positive |
| hsa-miR-708-3p | COL1A1 | 0.306 | 2.82E-10 | Positive |
| hsa-miR-708-5p | COL1A1 | 0.434 | 4.25E-20 | Positive |
| hsa-miR-1224-5p | CYP3A4 | 0.673 | 6.25E-55 | Positive |
| hsa-miR-184 | CYP3A4 | 0.708 | 5.47E-63 | Positive |
| hsa-miR-190b-5p | CYP3A4 | 0.373 | 7.23E-15 | Positive |
| hsa-miR-592 | CYP3A4 | 0.921 | 2.50E-167 | Positive |
| hsa-miR-210-3p | MYBL2 | 0.348 | 5.37E-13 | Positive |
| hsa-miR-18b-3p | RALYL | 0.301 | 5.83E-10 | Positive |
| hsa-miR-133b | SCN4A | 0.704 | 4.55E-62 | Positive |
| hsa-miR-675-3p | SCN4A | 0.492 | 3.84E-26 | Positive |

Table S3. MiRNAs related to which Redox_score they correlated with Redox_A and Redox_B.

|  | miRNAs | Coefficient | p-value | Regulation |
| --- | --- | --- | --- | --- |
| Redox_A | hsa-miR-133a-3p | -0.4105 | 9.62E-12 | Negative |
|  | hsa-miR-1-3p | -0.3940 | 7.33E-11 | Negative |
|  | hsa-miR-133b | -0.3743 | 7.23E-10 | Negative |
|  | hsa-miR-30a-3p | -0.3653 | 1.95E-09 | Negative |
|  | hsa-miR-1-5p | -0.3521 | 7.93E-09 | Negative |
|  | hsa-miR-133a-5p | -0.3438 | 1.86E-08 | Negative |
|  | hsa-miR-221-3p | -0.3129 | 3.58E-07 | Negative |
|  | hsa-miR-183-3p | 0.3002 | 1.10E-06 | Positive |
|  | hsa-miR-301b-3p | 0.3174 | 2.36E-07 | Positive |
|  | hsa-miR-940 | 0.3262 | 1.04E-07 | Positive |
|  | hsa-miR-210-3p | 0.3294 | 7.71E-08 | Positive |
| Redox_B | hsa-miR-133a-5p | -0.2725 | 6.04E-04 | Negative |
|  | hsa-miR-106a-3p | -0.3181 | 5.51E-05 | Negative |
|  | hsa-miR-222-3p | -0.2903 | 2.48E-04 | Negative |
|  | hsa-miR-499a-5p | -0.2814 | 3.89E-04 | Negative |
|  | hsa-miR-1-3p | -0.2936 | 2.09E-04 | Negative |
|  | hsa-miR-30a-5p | -0.2992 | 1.56E-04 | Negative |
|  | hsa-miR-505-3p | -0.2994 | 1.54E-04 | Negative |
|  | hsa-miR-363-5p | -0.2696 | 6.92E-04 | Negative |
|  | hsa-miR-1291 | -0.2799 | 4.21E-04 | Negative |
|  | hsa-miR-133b | -0.2841 | 3.40E-04 | Negative |
|  | hsa-miR-708-3p | 0.2904 | 2.47E-04 | Positive |
|  | hsa-miR-135a-5p | -0.2768 | 4.90E-04 | Negative |
|  | hsa-miR-30c-2-3p | -0.2799 | 4.19E-04 | Negative |
|  | hsa-miR-133a-3p | -0.3295 | 2.84E-05 | Negative |
|  | hsa-miR-381-5p | -0.2873 | 2.89E-04 | Negative |
